# Exploring the value of arterial spin labeling and six diffusion MRI models in differentiating solid benign and malignant renal tumors

**DOI:** 10.1186/s41747-024-00537-y

**Published:** 2024-12-05

**Authors:** Mengmeng Gao, Shichao Li, Guanjie Yuan, Weinuo Qu, Kangwen He, Zhouyan Liao, Ting Yin, Wei Chen, Qian Chu, Zhen Li

**Affiliations:** 1 Department of Radiology, Tongji Hospital, Tongji Medical College, Huazhong University of Science and Technology, Wuhan, China; 2 MR Research Collaboration Team, Siemens Healthineers Ltd, Chengdu, China; 3 MR Research Collaboration Team, Siemens Healthineers Ltd, Wuhan, China; 4 Department of Oncology, Tongji Hospital, Tongji Medical College, Huazhong University of Science and Technology, Wuhan, China

**Keywords:** Arterial spin labeling, Diagnosis (differential), Diffusion MRI, Kidney neoplasms, Magnetic resonance imaging

## Abstract

**Objective:**

To explore the value of three-dimensional arterial spin labeling (ASL) and six diffusion magnetic resonance imaging (MRI) models in differentiating solid benign and malignant renal tumors.

**Methods:**

This retrospective study included 89 patients with renal tumors. All patients underwent ASL and ZOOMit diffusion-weighted imaging (DWI) examinations and were divided into three groups: clear cell renal cell carcinoma (ccRCC), non-ccRCC, and benign renal tumors (BRT). The mean and peak renal blood flow (RBFmean and RBFpeak) from ASL and fourteen diffusion parameters from mono-exponential DWI (Mono_DWI), intravoxel incoherent motion (IVIM), diffusion kurtosis imaging (DKI), stretched exponential model (SEM), fractional order calculus (FROC), and continuous-time random-walk (CTRW) model were analyzed. Binary logistic regression was used to determine the optimal parameter combinations. The diagnostic performance of various MRI-derived parameters and their combinations was compared.

**Results:**

Among the six diffusion models, the SEM model achieved the highest performance in differentiating ccRCC from non-ccRCC (area under the receiver operating characteristic curve [AUC] 0.880) and from BRT (AUC 0.891). IVIM model achieved the highest AUC (0.818) in differentiating non-ccRCC from BRT. Among all the MRI-derived parameters, RBFpeak combined with DKI_MK yielded the highest AUC (0.970) in differentiating ccRCC from non-ccRCC, and the combination of RBFpeak, SEM_DDC, and FROC_μ yielded the highest AUC (0.992) for differentiating ccRCC from BRT.

**Conclusion:**

ASL and all diffusion models showed similar diagnostic performance in differentiating ccRCC from non-ccRCC or BRT, while the IVIM model performed better in distinguishing non-ccRCC from BRT. Combining ASL with diffusion models can provide additional value in predicting ccRCC.

**Relevance statement:**

Considering the increasing detection rate of incidental renal masses, accurate discrimination of benign and malignant renal tumors is crucial for decision-making. Combining ASL with diffusion MRI models offers a promising solution to this clinical issue.

**Key Points:**

- All assessed models were effective for differentiating ccRCC from non-ccRCC or BRT.
- ASL and all diffusion models showed similar performance in differentiating ccRCC from non-ccRCC or BRT.
- Combining ASL with diffusion models significantly improved diagnostic efficacy in predicting ccRCC.
- IVIM model could better differentiate non-ccRCC from BRT.

**Graphical Abstract:**

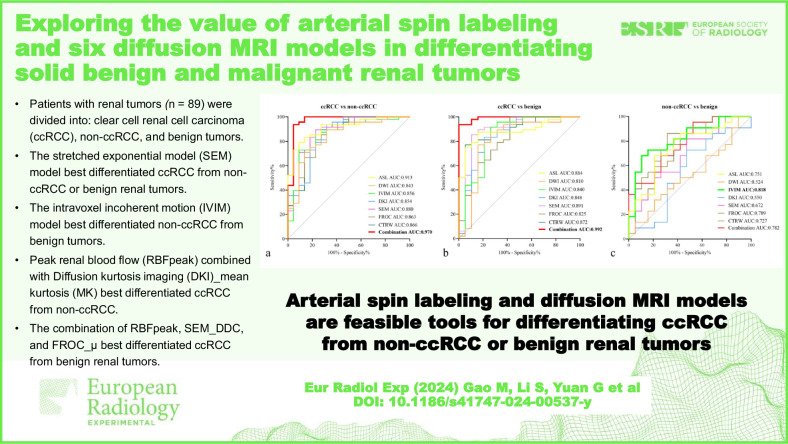

## Background

With the development of cross-sectional imaging, the detection rate of incidental renal masses has increased significantly [1, 2]. Most small renal masses (≤ 4 cm, T1a stage) are ultimately diagnosed as renal cell carcinoma (RCC), but up to 20% are benign, with oncocytoma and fat-poor angiomyolipoma (fpAML) being the most common [3, 4]. Even if malignant, most T1a RCCs are usually indolent and rarely progress locally or metastasize [5]. Recently, as an initial management option for elderly and comorbid patients with small renal masses, active surveillance has become increasingly accepted [6]. Therefore, it is crucial to accurately distinguish benign and malignant renal tumors and their biological behavior.

Percutaneous renal biopsy is considered the reference standard for preoperative histopathological diagnosis. However, it has certain limitations, including an approximately 10–15% non-diagnostic result and a 10% misdiagnosis. Moreover, not all renal masses are suitable for biopsy [7, 8]. As many renal masses are discovered incidentally during routine imaging examinations, image-based “virtual biopsy” may become an alternative for preoperative tumor characterization [9].

Diffusion-weighted imaging (DWI) is a magnetic resonance imaging (MRI) technique that can provide qualitative and quantitative information about a lesion by metrics based on the Brownian motion of water molecules in the tissue, which has been widely explored for tumor differential diagnosis and grading in previous retrospective researches [10–12]. However, the monoexponential DWI model, based on the Gaussian diffusion assumption, fails to represent actual water molecule diffusion. Research indicates that specific portions of the *b*-value ‘spectrum’ can reveal different diffusion properties [13]. The intravoxel incoherent motion (IVIM) model, utilizing a lower *b*-values spectrum (0–200, 800 s/mm^2^), helps distinguish between pure water molecular diffusion and microcirculatory perfusion [14]. Additionally, diffusion kurtosis imaging (DKI), stretched exponential model (SEM), fractional-order calculus model (FROC), and continuous-time random walk model (CTRW), which use higher *b*-values (1,000–4,000 s/mm^2^), address the non-Gaussian diffusion characteristics and provide insights into water diffusion heterogeneity from different dimensions, including time and space.

Previous studies have shown that the IVIM, DKI, and SEM models are valuable tools for renal tumor differential diagnosis and clear cell RCC (ccRCC) grading [12, 15]. Nevertheless, to date, no studies have used FROC and CTRW for the differential diagnosis of renal tumors. Several studies have assessed the efficacy of multiple diffusion models in differentiating benign and malignant renal tumors, but they have compared at most three or four models [10, 12, 15]. To our knowledge, no study has compared all DWI models within a single cohort of patients.

However, diffusion MRI models only represent single-dimension of tumor progression. Arterial spin labeling (ASL) MRI uses blood as an endogenous contrast agent to quantify tissue blood perfusion information [16, 17], which eliminates the potential risk of nephrogenic systemic fibrosis caused by gadolinium-base contrast agents. Compared to dynamic contrast-enhanced MRI, ASL can directly measure tissue perfusion and is relatively less affected by vascular permeability [18]. Lanzman et al [19] have demonstrated the clinical feasibility of ASL in noninvasive renal mass characterization, but their study was primarily based on single-slice two-dimensional pseudo-continuous ASL, with lower image signal-to-noise ratio, limited coverage, and more magnetic susceptibility artifacts. The three-dimensional readout method offers a higher signal-to-noise ratio due to superior background suppression and off-resonance artifact insensitivity [20]. However, research on employing three-dimensional pseudocontinuous ASL to distinguish benign and malignant renal tumors is still lacking.

Therefore, our study aimed to: (1) evaluate the clinical value of six diffusion MRI models in distinguishing solid benign and malignant renal tumors and compare their diagnostic performances, and (2) assess whether combining ASL with diffusion MRI models could improve the renal tumors’ diagnostic accuracy.

## Methods

### Patients

**Fig. 1:**
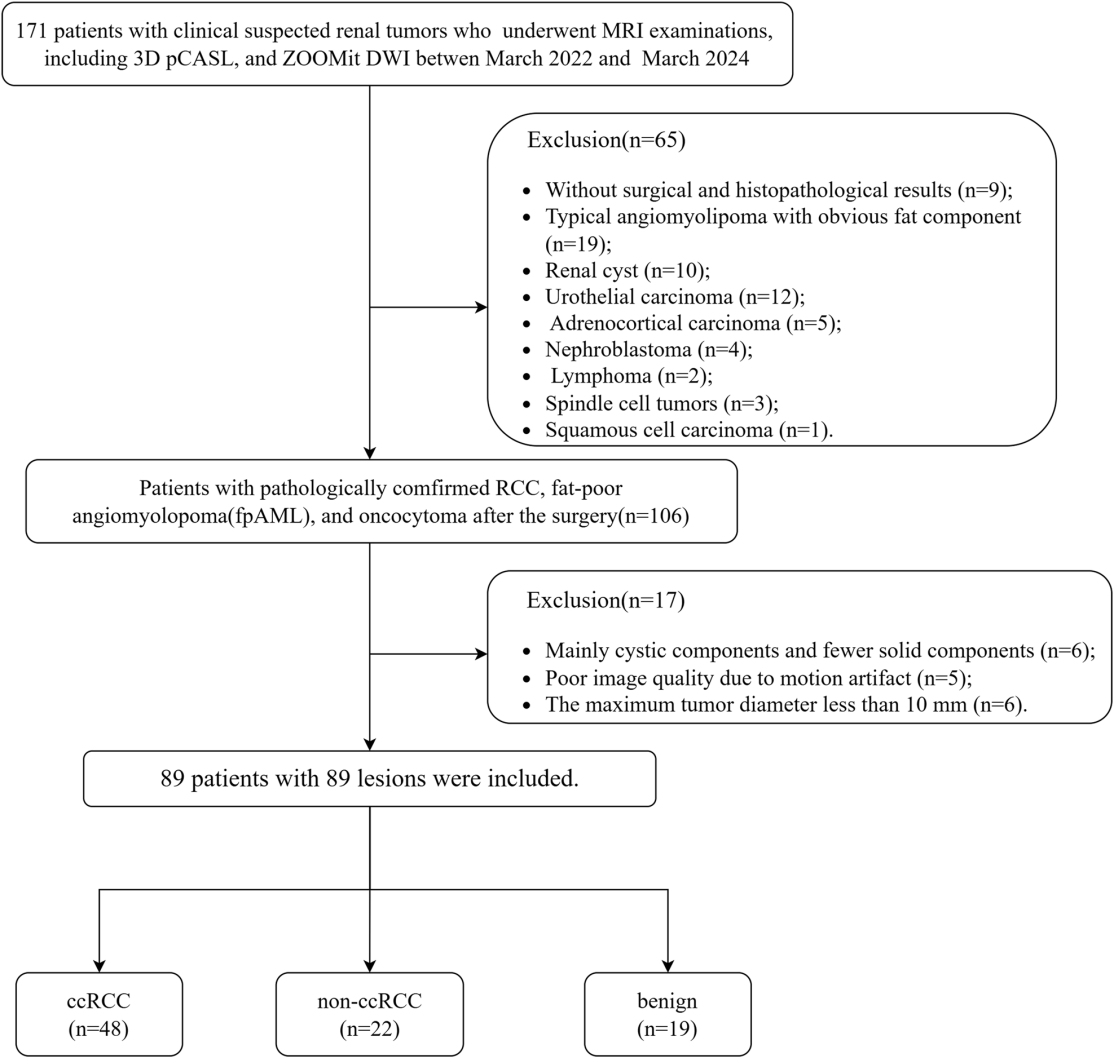
Flow chart for the selection of participants

The Ethics Committee of our hospital approved this retrospective study, and the requirement for informed consent was waived. From March 2022 to March 2024, 171 consecutive patients with or suspected of having renal tumors underwent MRI examinations based on previous ultrasound or computed tomography examinations, including ASL, and ZOOMit DWI sequences. The exclusion criteria were as follows: (1) no surgical and histopathological results (*n* = 9); (2) typical angiomyolipoma with obvious fat component (*n* = 19); (3) pathologically confirmed tumors other than RCC, fpAMLs, and oncocytoma, including the following: renal cysts (*n* = 10), urothelial carcinoma (*n* = 12), adrenocortical carcinoma (*n* = 5), nephroblastoma (*n* = 4), lymphoma (*n* = 2), spindle cell tumors (*n* = 3), squamous cell carcinoma (*n* = 1); (4) mainly cystic components and fewer solid components (*n* = 6); (5) poor image quality due to motion artifact (*n* = 5); and (6) maximum tumor diameter < 10 mm (*n* = 6). Finally, 89 patients with 89 lesions were included. The flow chart of the patient selection is shown in Fig. 1.

### MRI protocol

A 3-T MRI scanner (Magnetom Skyra, Siemens Healthcare, Erlangen, Germany) with an 18-channel body phased-array coil was used. Conventional scan sequences include coronal T2-weighted and axial T1-weighted and T2-weighted sequences.

#### ZOOMit DWI

Diffusion-weighted MRI was acquired using a research sequence with a reduced (“zoomed”) field of view (ZOOMit Pro, Siemens Healthineers). Compared to traditional DWI sequences, ZOOMit DWI applies both echo-planar imaging and another parallel radiofrequency pulse sequence to obtain a zoomed field of view, which allows for better image quality and provides more anatomical detail. To overcome motion artifacts, the images were processed with in-plane registration and complex averaging [21, 22], which provides a sufficient signal-to-noise ratio for diffusion images, and allows for free-breathing scans. ZOOMit DWI supports parallel acquisition technique reference scanning, and generalized auto-calibrating partial parallel acquisition−GRAPPA was employed, offering shorter scan times and higher temporal and/or spatial resolution. ZOOMit DWI with 11 *b*-values were performed with the following parameters: repetition time/echo time = 7,700/71 ms; fat saturation with spectral presaturation with inversion-recovery; field of view 288 × 125 mm; pixel size 1.2 × 1.2 mm^2^; slice thickness 4 mm; slice gap 1 mm; bandwidth 1,666 Hz; *b*-values 0 (1) s/mm^2^, 20 (1) s/mm^2^, 50 (1) s/mm^2^, 80 (1) s/mm^2^, 100 (1) s/mm^2^, 200 (1) s/mm^2^, 500 (1) s/mm^2^, 800 (2) s/mm^2^, 1,000 (2) s/mm^2^, 1,500 (3) s/mm^2^, and 2,000 (6) s/mm^2^. Acquisition time was 10:16 min:s.

#### ASL

ASL was performed with a research sequence developed by Siemens Healthineers for renal pseudo-continuous ASL, which provided a free-breathing three-dimensional turbo gradient spin echo sequence with optimized background suppression and arterial blood suppression [23, 24]. In-plane motion correction was performed retrospectively using a two-dimensional elastic registration algorithm [24]. Sixteen pairs of control and label images were acquired with the following parameters: repetition time/echo time 5,000/19.28 ms; field of view 300 × 150 mm; voxel size 4.7 × 4.7 × 5.0 mm^2^; number of slice 16; bandwidth 3,552 Hz. Acquisition time was 3:25 min:s.

### Image analysis

Multiple *b*-value ZOOMit DWI images were analyzed using the postprocessing software Body-DiffusionLab (BoDiLab, Chengdu ZhongYing Medical Technology Co., Ltd., Chengdu, China) to generate six diffusion models. The corresponding formulas for each model are presented in Supplemental Appendix S1.

Totally, 14 parameters were derived from the six diffusion models (Mono_ADC, IVIM_D, IVIM_D*, IVIM_f, DKI_MD, DKI_MK, SEM_α, SEM_DDC, FROC_β, FROC_μ, FROC_D, CTRW_α, CTRW_β, CTRW_D). After data acquisition, quantitative renal blood flow (RBF) maps were developed inline, pixel by pixel, using the formula presented in Supplemental Appendix S2.

All diffusion metrics and the maximum tumor diameter were measured independently by two radiologists (with 3 years and 6 years of experience in renal MRI, respectively), who were blinded to the pathological diagnosis. The maximal diameter of the tumor was measured on axial T2-weighted images. To measure the diffusion MRI-derived parameters, free-hand regions of interest (ROI) were outlined around the tumor on the apparent diffusion coefficient (ADC) quantitative map and automatically copied to the other 13 quantitative maps using the copy-and-paste method on ITK-SNAP. To minimize selection bias, the largest possible ROI that included the largest cross-sectional of the tumor was delineated using T2-weighted images as a reference. To measure the ASL parameters, the mean perfusion level was measured with a ROI drawn around the outer contour of the largest tumor cross-section. Additionally, a ROI of approximately 1 cm² was placed in the visually highest signal intensity regions on the RBF maps to evaluate the peak tumor perfusion. All pathological results were reviewed by an experienced senior pathologist.

### Statistical analysis

GraphPad Prism (version 9.0), MedCalc (version 20.0), and SPSS (version 25.0) were used for all statistical analysis. The interobserver agreement of each parameter was estimated using the intraclass correlation coefficients (> 0.80, excellent; 0.61–0.80, good; 0.41–0.60, moderate; 0.21–0.40, fair; and ≤ 0.20, poor). The normality of continuous variables was assessed using the Shapiro–Wilk test. Continuous variables presented as mean ± standard deviation were compared using one-way ANOVA (Tukey’s test) or the Kruskal–Wallis *H*-test (with Bonferroni correction) among the ccRCC, non-ccRCC, and benign renal tumors. Categorical variables presented as a number of cases (%) were compared with the chi-square test. Univariate logistic regression analysis was used for the initial MRI parameters selection. Subsequently, a forward-stepwise multivariate logistic regression analysis was performed on parameters with *p* < 0.05 to determine the optimal combination of parameters for discriminating ccRCC from non-ccRCC or benign renal tumors, as well as for distinguishing non-ccRCC from benign renal tumors. Receiver operating characteristic curves were plotted, and the area under the receiver operating characteristic curve (AUCs) was obtained to quantify the discriminant ability. The DeLong test was used to compare the AUCs of different parameters and their combinations. The goodness-of-fit of the ASL and six diffusion MRI models was assessed using the Akaike information criterion (AIC) [25]. Statistical significance was defined as two-sided *p* < 0.05.

## Results

### Participant characteristics

A total of 89 patients with renal tumors were included in the study. Among them, 70 patients were confirmed to have malignant renal tumors, while 19 patients had benign renal tumors. Of the malignant renal tumors, 48 patients (mean age 55.9 ± 11.6 years) were diagnosed with ccRCC (World Health Organization/International Society of Urological Pathology: grade I, *n* = 10; grade II, *n* = 24; grade III, *n* = 10; grade IV, *n* = 4) while 22 patients (mean age 52.4 ± 14.7 years) had non-ccRCC, including papillary RCC (*n* = 4), chromophobe RCC (*n* = 12), unclassified RCC (*n* = 2), MiT family translocation RCC (*n* = 1), TFE3 translocation RCC (*n* = 1), TFE3 gene fusion-related RCC (*n* = 1), and Xp11.2 translocation/TFE3 gene fusion-related RCC (*n* = 1). The benign renal tumors (*n* = 19; mean age, 47.6 ± 14.9 years) consisted of oncocytomas (*n* = 8) and fpAML (*n* = 11). There was a higher proportion of females in the benign renal tumor group, compared to ccRCC and non-ccRCC groups (*p* = 0.011). The maximum tumor diameter of benign renal tumors was significantly smaller than those of ccRCC and non-ccRCC (*p* = 0.021). Furthermore, the age, BMI, and tumor location were not statistically different among the ccRCC, non-ccRCC, and benign renal tumor groups.

### Interobserver reliability assessment

With intraclass correlation coefficient values ranging from 0.920 to 0.993, the two radiologists’ interobserver agreements for the ASL and diffusion MRI-derived parameters were excellent, demonstrating good measurement repeatability and reproducibility (Table S2). Thus, for the final statistical analysis, the measurements from one of the radiologists were chosen at random.

### Comparison of quantitative MRI-derived parameters

**Fig. 2:**
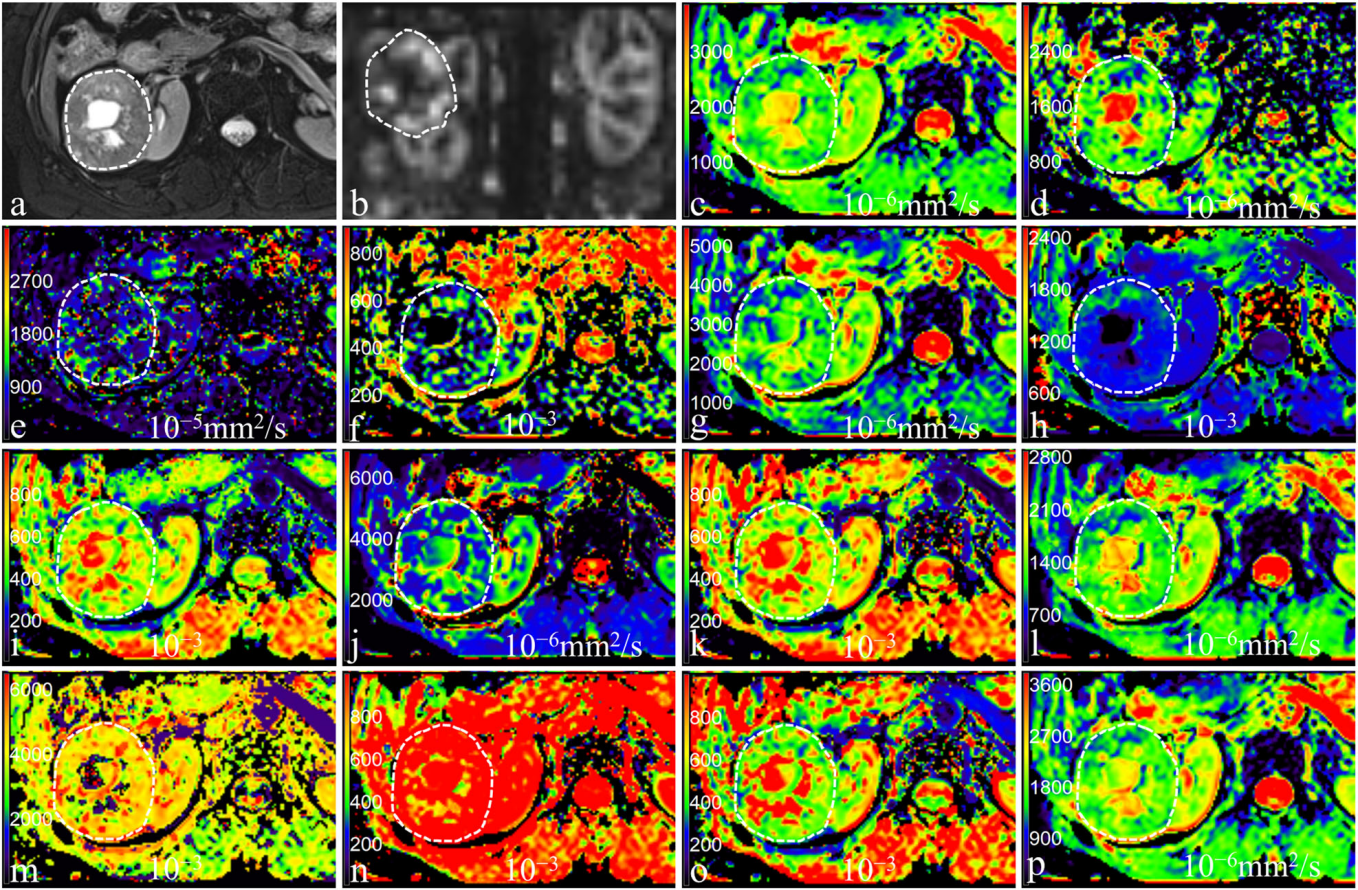
A 61-year-old woman with pathologically confirmed clear cell renal cell carcinoma in the right kidney. **a** Axial T2-weighted imaging shows a mixed-signal-intensity mass; **b** RBF map shows the perfusion level of mass, with the RBFmean and RBFpeak of 167.45 mL/100 g/min and 384.00 mL/100 g/min; **c**–**p** pseudo-colorized images showed the Mono_ADC (**c**), IVIM_D (**d**), IVIM_D* (**e**), IVIM_f (**f**), DKI_MD (**g**), DKI_MK (**h**), SEM_α (**i**), SEM_DDC (**j**), FROC_β (**k**), FROC_D (**l**), FROC_μ (**m**), CTRW_α (**n**), CTRW_β (**o**), and CTRW_D (**p**), with the values of 1.79 × 10^−3^ mm^2^/s, 1.52 × 10^−3^ mm^2^/s, 11.5 × 10^−3^ mm^2^/s, 20.96%, 2.25 × 10^−3^ mm^2^/s, 0.60 × 10^−3^, 0.63 × 10^−3^, 2.36 × 10^−3^ mm^2^/s, 0.67 × 10^−3^, 1.41 × 10^−3^ mm^2^/s, 4.03 × 10^−3^, 0.94 × 10^−3^, 0.64 × 10^−3^, and 1.87 × 10^−3^ mm^2^/s, respectively

**Fig. 3:**
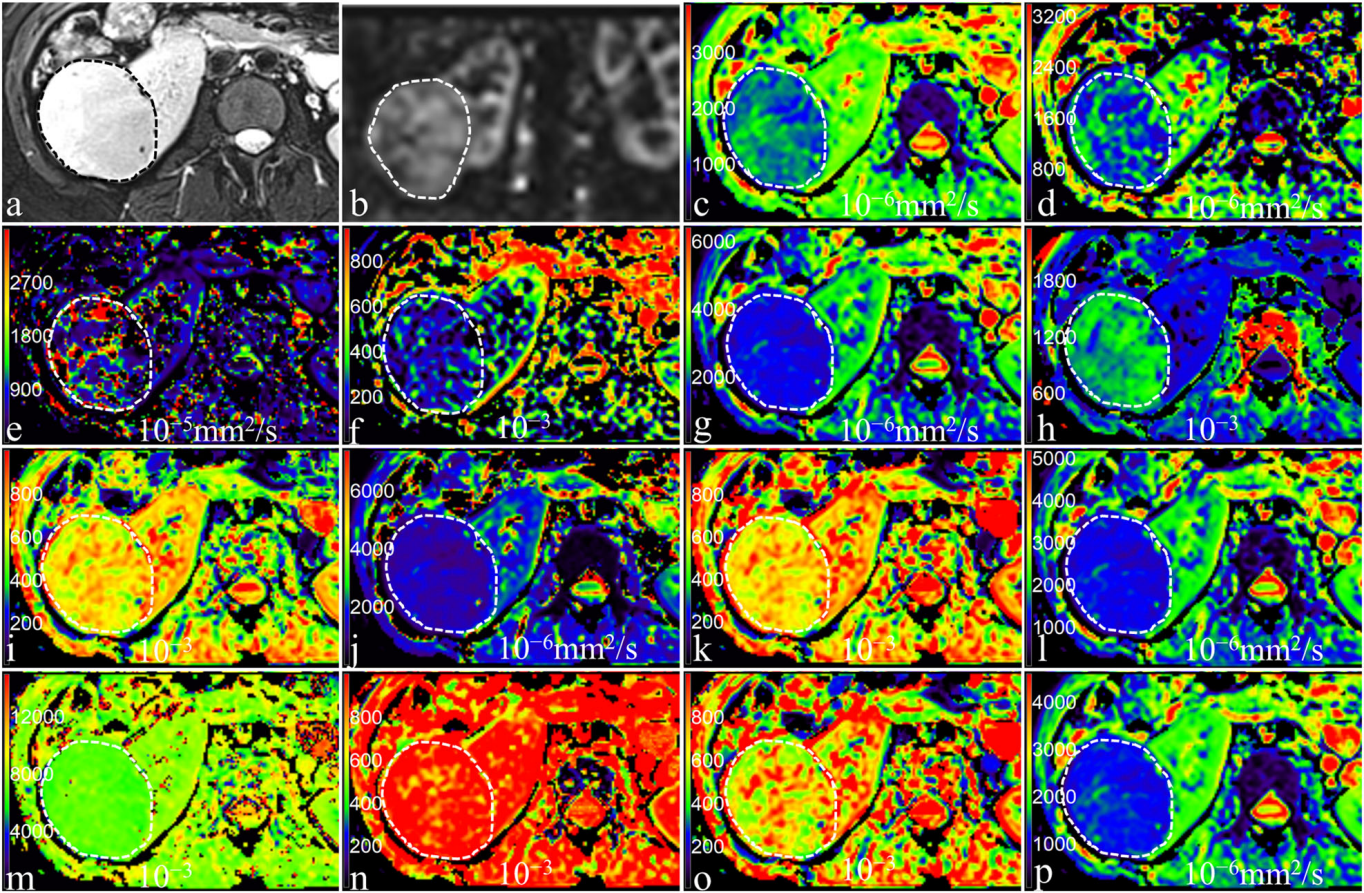
A 43-year-old woman with pathologically confirmed chromophobe renal cell carcinoma in the right kidney. **a** Axial T2-weighted imaging shows a moderate-signal-intensity mass; **b** RBF map shows the perfusion level of mass, with the RBFmean and RBFpeak of 156.13 mL/100 g/min and 272.00 mL/100 g/min; **c**–**p** pseudo-colorized images showed the Mono_ADC (**c**), IVIM_D (**d**), IVIM_D* (**e**), IVIM_f (**f**), DKI_MD (**g**), DKI_MK (**h**), SEM_α (**i**), SEM_DDC (**j**), FROC_β (**k**), FROC_D (**l**), FROC_μ (**m**), CTRW_α (**n**), CTRW_β (**o**), and CTRW_D (**p**), with the values of 1.15 × 10^−3^ mm^2^/s, 0.93 × 10^−3^ mm^2^/s, 11.82 × 10^−3^ mm^2^/s, 15.38%, 1.43 × 10^−3^ mm^2^/s, 0.85 × 10^−3^, 0.69 × 10^−3^, 1.17 × 10^−3^ mm^2^/s, 0.70 × 10^−3^, 0.85 × 10^−3^ mm^2^/s, 3.82 × 10^−3^, 0.93 × 10^−3^, 0.71 × 10^−3^, and 1.18 × 10^−3^ mm^2^/s, respectively

**Fig. 4:**
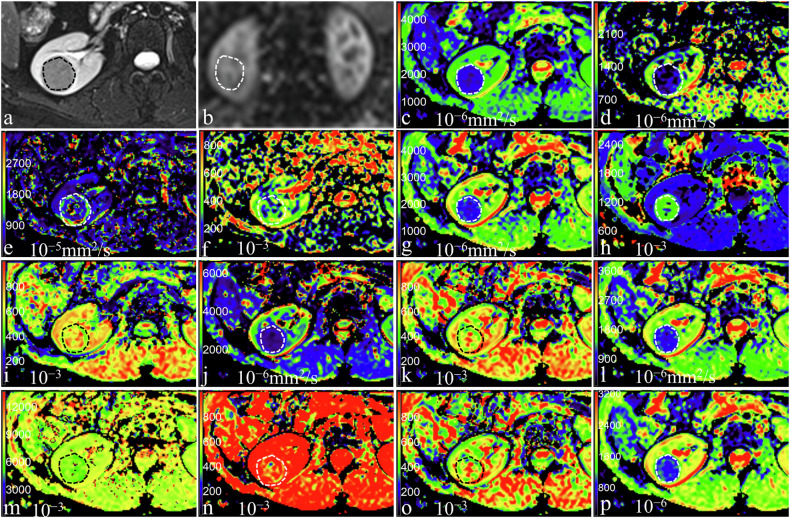
A 26-year-old woman with pathologically confirmed fpAML in the right kidney. **a** Axial T2-weighted imaging shows a low-signal-intensity mass; **b** RBF map shows the perfusion level of mass, with the RBFmean and RBFpeak of 190.56 mL/100 g/min and 244.00 mL/100 g/min; **c**–**p** pseudo-colorized images showed the Mono_ADC (**c**), IVIM_D (**d**), IVIM_D* (**e**), IVIM_f (**f**), DKI_MD (**g**), DKI_MK (**h**), SEM_α (**i**), SEM_DDC (**j**), FROC_β (**k**), FROC_D (**l**), FROC_μ (**m**), CTRW_α (**n**), CTRW_β (**o**), and CTRW_D (**p**), with the values of 1.07 × 10^−3^ mm^2^/s, 0.69 × 10^−3^ mm^2^/s, 11.56 × 10^−3^ mm^2^/s, 23.89%, 1.44 × 10^−3^ mm^2^/s, 0.98 × 10^−3^, 0.58 × 10^−3^, 0.97 × 10^−3^ mm^2^/s, 0.64 × 10^−3^, 1.04 × 10^−3^ mm^2^/s, 6.58 × 10^−3^, 0.88 × 10^−3^, 0.63 × 10^−3^, and 1.01 × 10^−3^ mm^2^/s, respectively

**Fig. 5:**
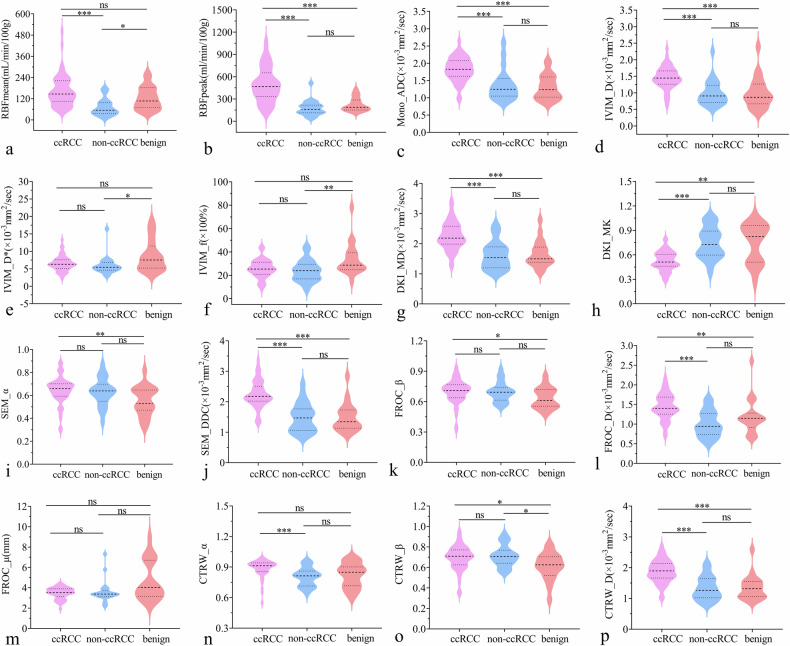
Violin graphs showing the significant quantitative parameters from ASL and six MRI diffusion models among the ccRCCs, non-ccRCCs, and benign renal tumors (**a**–**p**). The statistically significant level, ***p ≤ 0.001; **p < 0.025; *p < 0.05; ns, Not significant

Figures 2–4 shows the representative images of patients with pathologically confirmed ccRCC, non-ccRCC, and benign renal tumors, respectively. The ASL and diffusion-derived parameters of ccRCC, non-ccRCC, and benign renal tumors are shown in Fig. 5 and Table 1. The RBFmean, RBFpeak, Mono_ADC, IVIM_D, DKI_MD, SEM_DDC, FROC_D, CTRW_α, and CTRW_D in ccRCC were significantly higher, while DKI_MK was significantly lower compared to non-ccRCC (all *p* < 0.05). The RBFpeak, Mono_ADC, IVIM_D, DKI_MD, SEM_α, SEM_DDC, FROC_β, FROC_D, CTRW_β, and CTRW_D in ccRCC were higher, while DKI_MK was lower compared to benign renal tumors (all *p* < 0.05). The RBFmean, IVIM_D*, and IVIM_f in non-ccRCC were lower, while CTRW_β were higher compared to benign renal tumors (all *p* < 0.05). However, the other parameters did not show statistical significance between non-ccRCC and benign renal tumors (*p* > 0.05).

**Table 1:** Comparison of ASL and diffusion MRI-derived parameters among ccRCC, non-ccRCC, and benign renal tumors

	ccRCC, (*n* = 48)	Non-ccRCC, (*n* = 22)	Benign, (*n* = 19)	*p* value	*p* value		
					ccRCC vs non-ccRCC	ccRCC vs benign	Non-ccRCC vs benign
RBFmean	164.29 ± 91.35	71.83 ± 48.30	123.73 ± 65.85	*<* 0.001^*^	*<* 0.001^*^	0.348^*^	0.033^*^
RBFpeak	490.23 ± 234.89	175.53 ± 102.74	224.42 ± 91.87	*<* 0.001^*^	*<* 0.001^*^	*<* 0.001^*^	0.771^*^
Mono_ADC	1.83 ± 0.35	1.31 ± 0.36	1.37 ± 0.46	*<* 0.001^*^	*<* 0.001^*^	*<* 0.001^*^	1.000^*^
IVIM_D	1.45 ± 0.34	0.99 ± 0.39	1.00 ± 0.48	*<* 0.001^*^	*<* 0.001^*^	*<* 0.001^*^	1.000^*^
IVIM_D^*^	6.51 ± 2.22	5.94 ± 2.68	8.68 ± 4.51	0.049^*^	0.347^*^	0.554^*^	0.044^*^
IVIM_f	25.65 ± 7.97	23.83 ± 9.29	33.91 ± 14.10	0.015^*^	1.000^*^	0.062^*^	0.016^*^
DKI_MD	2.26 ± 0.47	1.61 ± 0.46	1.66 ± 0.44	*<* *0.001*	*<* *0.001*	*0.001*	0.951
DKI_MK	0.52 ± 0.12	0.75 ± 0.19	0.75 ± 0.24	*<* *0.001*	*<* *0.001*	*0.003*	1.000
SEM_α	0.64 ± 0.12	0.63 ± 0.12	0.55 ± 0.13	0.028^*^	1.000	*0.022*	0.249
SEM_DDC	2.24 ± 0.45	1.48 ± 0.48	1.48 ± 0.47	*<* 0.001^*^	*<* 0.001^*^	*<* 0.001^*^	1.000^*^
FROC_β	0.70 ± 0.12	0.70 ± 0.10	0.63 ± 0.10	0.044^*^	1.000^*^	0.038^*^	0.306^*^
FROC_D	1.45 ± 0.33	1.00 ± 0.33	1.20 ± 0.46	*<* 0.001^*^	*<* 0.001^*^	0.009^*^	0.683^*^
FROC_μ	3.48 ± 0.56	3.59 ± 1.09	4.66 ± 1.95	0.101^*^	NA	NA	NA
CTRW_α	0.89 ± 0.09	0.80 ± 0.09	0.82 ± 0.12	0.001^*^	0.001^*^	0.065^*^	1.000^*^
CTRW_β	0.69 ± 0.12	0.71 ± 0.11	0.61 ± 0.13	*0.020*	0.834	*0.037*	*0.026*
CTRW_D	1.88 ± 0.37	1.33 ± 0.39	1.34 ± 0.40	*<* 0.001^*^	*<* 0.001^*^	*<* 0.001^*^	1.000^*^

Data are expressed as mean ± standard deviation (SD)

The *p* values in italics are of statistical significance

The unit for “RBF” is mL/100 g/min. DKI_MK, SEM_α, FROC_β, FROC_μ, CTRW_α, CTRW_β have no unit. Mono_ADC, IVIM_D, IVIM_D*, DKI_MD, SEM_DDC, FROC_D, CTRW_D values are presented in × 10^−3^ mm^2^/s, IVIM_f is presented in percentage

*NA* Not applicable (there was no statistical difference using the Kruskal–Wallis *H*-test, so Bonferroni correction pairwise comparisons were not performed), *ADC* Apparent diffusion coefficient, *CTRW_α* Temporal diffusion heterogeneity index, *CTRW_β* Spatial diffusion heterogeneity index, *DKI_MD* Mean diffusivity, *DKI_MK* Mean kurtosis, *FROC_β* Spatial diffusion heterogeneity index, *FROC_μ* Spatial diffusion constant, *IVIM_D* True diffusivity, IVIM_D* Pseudo-diffusion coefficient, *IVIM_f* Perfusion fraction, *RBF* Renal blood flow, *SEM_DDC*· *FROC_D· CTRW_D* Diffusion coefficient, *SEM_α* Intravoxel heterogeneity index

^*^ Kruskal–Wallis *H*-test with Bonferroni correction, others are one-way ANOVA

### Diagnostic performance of multiple parameters

**Fig. 6:**
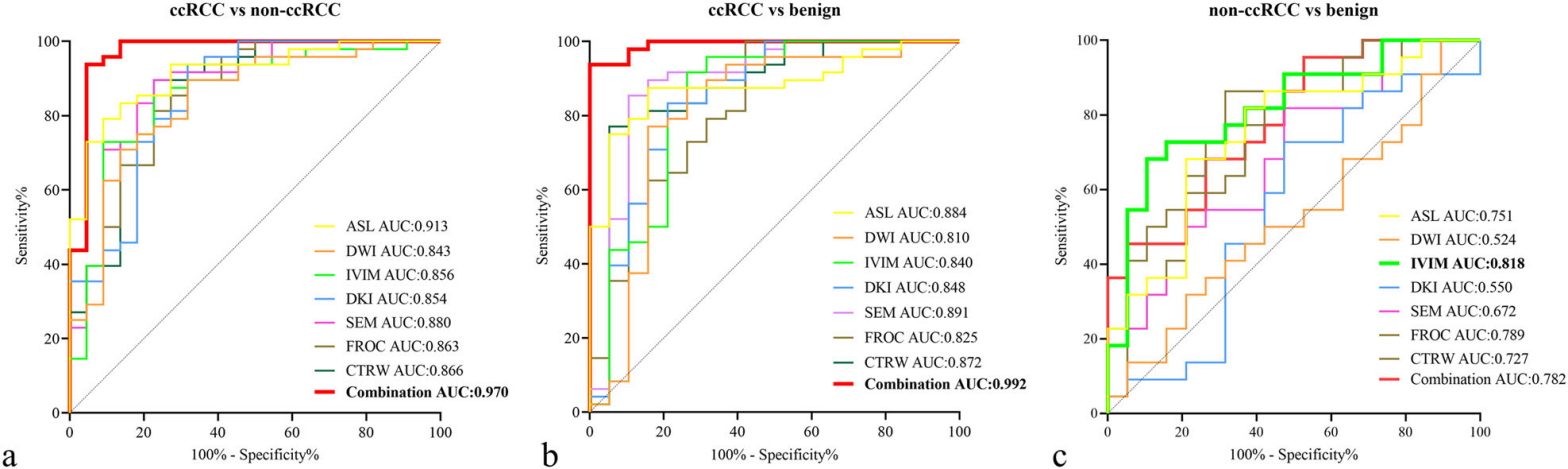
Receiver operating characteristic (ROC) curves of ASL, six diffusion MRI models, and their combinations for discrimination benign and malignant renal tumors. **a** Differentiation of ccRCC from non-ccRCC. The combined model comprises RBFpeak and DKI_MK. **b** Differentiation of ccRCC from benign renal tumors. The combined model comprises RBFpeak, SEM_DDC, and FROC_μ. **c** Differentiation of non-ccRCC from benign renal tumors. The combined model comprises RBFmean and IVIM_f

Receiver operating characteristic analysis of ASL and multiple diffusion-derived parameters, as well as their combinations for differentiating ccRCC from non-ccRCC or benign renal tumors, and non-ccRCC from benign renal tumors, are presented in Fig. 6 and Tables 2–4.

**Table 2:** ROC analyzes ASL and diffusion parameters in discriminating ccRCC from non-ccRCC

	AUC (95% CI)	Cut-off value	Sensitivity	Specificity	*p*	*p* for comparison	AIC
RBFmean	0.847 (0.741–0.922)	100.08	77.08	81.82	< 0.001^*^	0.024^*^	–
RBFpeak	0.907 (0.813–0.963)	279.33	79.17	95.45	< 0.001^*^	0.023^*^	–
Mono_ADC	0.843 (0.736–0.919)	1.388	89.58	68.18	< 0.001^*^	0.005^*^	65.083
IVIM_D	0.837 (0.730–0.915)	1.136	87.50	72.73	< 0.001^*^	0.029^*^	–
IVIM_D^*^	0.627 (0.503–0.740)	6.029	60.42	72.73	0.077	< 0.001^*^	–
IVIM_f	0.577 (0.453–0.694)	18.220	85.42	36.36	0.328	< 0.001^*^	–
DKI_MD	0.838 (0.731–0.915)	1.920	79.17	81.82	< 0.001^*^	0.006^*^	–
DKI_MK	0.835 (0.727–0.913)	0.645	87.50	63.64	< 0.001^*^	0.013^*^	–
SEM_α	0.553 (0.429–0.672)	0.669	45.83	72.73	0.488	< 0.001^*^	–
SEM_DDC	0.883 (0.783–0.947)	1.175	91.67	77.27	< 0.001^*^	0.018^*^	–
FROC_β	0.556 (0.432–0.675)	0.726	45.83	77.27	0.460	< 0.001^*^	–
FROC_D	0.835 (0.727–0.913)	1.019	91.67	63.64	< 0.001^*^	0.003^*^	–
FROC_μ	0.580 (0.456–0.697)	3.425	64.58	68.18	0.296	< 0.001^*^	–
CTRW_α	0.773 (0.657–0.864)	0.844	83.33	72.73	< 0.001^*^	0.001^*^	–
CTRW_β	0.511 (0.389–0.633)	0.850	93.75	18.18	0.881	< 0.001^*^	–
CTRW_D	0.848 (0.742–0.922)	1.365	93.75	63.64	< 0.001^*^	0.005^*^	–
ASL	0.913 (0.821–0.967)	–	79.17	90.91	< 0.001^*^	0.042^*^	54.868
IVIM	0.856 (0.752–0.929)	–	72.92	90.91	< 0.001^*^	0.015^*^	63.036
DKI	0.854 (0.749–0.927)	–	93.75	68.18	< 0.001^*^	0.013^*^	60.164
SEM	0.880 (0.780–0.945)	–	89.58	77.27	< 0.001^*^	0.015^*^	60.544
FROC	0.863 (0.759–0.933)	–	81.25	77.27	< 0.001^*^	0.012^*^	63.548
CTRW	0.866 (0.764–0.936)	–	89.58	77.27	< 0.001^*^	0.017^*^	65.266
Combination	0.970 (0.898–0.996)	–	93.75	95.45	< 0.001^*^	Ref	31.832

*AIC* Akaike information criterion, *AUC* Area under the curve, *CI* Confidence interval, *Ref* Reference

Combination, RBFpeak + DKI_MK

^*^ The *p* values are of statistical significance

**Table 3:** ROC analyzes ASL and diffusion parameters in discriminating ccRCC from benign renal tumors

	AUC (95% CI)	Cut-off value	Sensitivity	Specificity	*p*	*p* for comparison	AIC
RBFmean	0.634 (0.507–0.748)	107.18	72.92	52.63	0.087	< 0.001^*^	–
RBFpeak	0.854 (0.746–0.928)	322	77.08	84.21	< 0.001^*^	0.002^*^	–
Mono_ADC	0.810 (0.696–0.896)	1.617	77.08	84.21	< 0.001^*^	0.011^*^	67.157
IVIM_D	0.810 (0.696–0.896)	1.039	89.58	68.42	< 0.001^*^	0.008^*^	–
IVIM_D^*^	0.615 (0.488–0.732)	7.949	83.33	47.37	0.186	< 0.001^*^	–
IVIM_f	0.688 (0.563–0.795)	23.596	39.58	94.74	0.010^*^	< 0.001^*^	–
DKI_MD	0.848 (0.739–0.924)	0.708	83.33	78.95	< 0.001^*^	0.007^*^	–
DKI_MK	0.762 (0.642–0.858)	0.701	95.83	63.16	0.001^*^	0.004^*^	–
SEM_α	0.711 (0.587–0.815)	0.529	85.42	52.63	0.002^*^	< 0.001^*^	–
SEM_DDC	0.886 (0.785–0.951)	1.735	89.58	84.21	< 0.001^*^	0.051	–
FROC_β	0.686 (0.561–0.794)	0.610	83.33	52.63	0.011^*^	< 0.001^*^	–
FROC_D	0.740 (0.618–0.840)	1.190	81.25	73.68	0.001^*^	< 0.001^*^	–
FROC_μ	0.645 (0.518–0.758)	4.011	87.50	52.63	0.105	< 0.001^*^	–
CTRW_α	0.671 (0.545–0.781)	0.901	56.25	78.95	0.044^*^	< 0.001^*^	–
CTRW_β	0.686 (0.561–0.794)	0.705	54.17	78.95	0.011^*^	< 0.001^*^	–
CTRW_D	0.740 (0.618–0.840)	1.190	81.25	73.68	0.001^*^	0.024^*^	–
ASL	0.884 (0.782–0.949)	–	87.50	84.21	< 0.001^*^	0.008^*^	56.361
IVIM	0.840 (0.730–0.918)	–	91.67	73.68	< 0.001^*^	0.020^*^	65.638
DKI	0.848 (0.739–0.924)	–	83.33	78.95	< 0.001^*^	0.018^*^	59.059
SEM	0.891 (0.791–0.954)	–	85.42	89.47	< 0.001^*^	0.067	55.087
FROC	0.825 (0.721–0.907)	–	100.00	57.89	< 0.001^*^	0.005^*^	63.674
CTRW	0.872 (0.767–0.941)	–	77.08	94.74	< 0.001^*^	0.033^*^	62.709
Combination	0.992 (0.932–1.000)	–	93.75	100.00	< 0.001^*^	Ref	21.003

*AIC* Akaike information criterion, *AUC* Area under the curve, *CI* Confidence interval, *Ref* Reference

Combination, RBFpeak + SEM_DDC + FROC_μ

^*^ The *p* values are of statistical significance

**Table 4:** ROC analyzes ASL and diffusion parameters in discriminating non-ccRCC from benign renal tumors

	AUC (95% CI)	Cut-off value	Sensitivity	Specificity	*p*	*p* for comparison	AIC
RBFmean	0.754 (0.594–0.874)	97.23	77.27	63.16	< 0.001^*^	0.467	
IVIM_D^*^	0.699 (0.535–0.832)	7.149	86.36	57.89	0.023^*^	0.113	
IVIM_f	0.742 (0.581–0.865)	23.569	50.00	94.74	0.002^*^	0.252	
CTRW_β	0.703 (0.540–0.836)	0.637	77.27	57.89	0.014^*^	0.149	
ASL	0.751 (0.592–0.873)	–	68.18	78.95	0.001^*^	0.481	54.466
Mono_ADC	0.524 (0.362–0.682)	1.035	31.82	78.95	0.797	0.001^*^	60.327
IVIM	0.818 (0.667–0.921)	–	68.18	89.47	< 0.001^*^	Ref	51.832
DKI	0.550 (0.387–0.706)	–	72.73	52.63	0.601	0.002^*^	62.434
SEM	0.672 (0.508–0.810)	–	81.82	52.63	0.044^*^	0.062	58.430
FROC	0.789 (0.634–0.901)	–	86.36	68.42	< 0.001^*^	0.675	52.737
CTRW	0.727 (0.566–0.854)	–	77.27	63.16	0.005^*^	0.170	56.850
Combination	0.782 (0.626–0.896)	–	95.45	47.37	< 0.001^*^	0.596	51.169

*AIC* Akaike information criterion, *AUC* Area under the curve, *CI* Confidence interval, *Ref* Reference

Combination, RBFmean + IVIM_f

^*^ The *p* values are of statistical significance

As for differentiating ccRCC from non-ccRCC, the AUCs of RBFmean, RBFpeak, and 14 diffusion-derived parameters ranged from 0.511 (95% confidence interval [CI]: 0.389–0.633) to 0.907 (95% CI: 0.813–0.963), with sensitivities ranging from 45.8% to 93.8% and specificities ranging from 18.2% to 95.5%. Among ASL and the six diffusion models, ASL achieved the highest AUC of 0.913 (95% CI: 0.821–0.967), followed by the SEM model, which had an AUC of 0.880 (95% CI: 0.780–0.945). Furthermore, the combination of RBFpeak and DKI_MK achieved the highest AUC of 0.970 (95% CI: 0.898–0.996) and the lowest AIC of 31.832, with the AUC of the combination being significantly higher compared to ASL or any of the diffusion models alone (all *p* < 0.05).

In terms of differentiating ccRCC from benign renal tumors, the AUCs of RBFmean, RBFpeak, and 14 diffusion-derived parameters ranged from 0.615 (95% CI: 0.488–0.732) to 0.886 (95% CI: 0.785–0.951), with sensitivities ranging from 39.6% to 95.8%, specificities ranging from 52.6% to 94.7%. Among ASL and the six diffusion models, the SEM model yielded the highest AUC of 0.891 (95% CI: 0.791–0.954), although there was no statistical difference compared to ASL and other diffusion models (all *p* > 0.05). Based on the results of logistic regression analysis, RBFpeak, SEM_DDC, and FROC_μ were included in the combination with the highest AUC of 0.992 (95% CI: 0.932–1.000) and the lowest AIC of 21.003. Moreover, the AUC of the combination was significantly higher than those of ASL and any of the diffusion models except for the SEM model (*p* < 0.05).

Regarding the differentiation of non-ccRCC and benign renal tumors, the AUCs of RBFmean, IVIM_D*, IVIM_f, and CTRW_β were 0.754, 0.699, 0.742, and 0.703. Among ASL and the six diffusion models, the IVIM model yielded the highest AUC of 0.818 (95% CI: 0.667–0.921), which was significantly higher than those of the Mono_ADC and DKI models (*p* < 0.05). The combination of RBFmean and IVIM_f yielded an AUC of 0.782, with a significant difference compared to DKI (*p* = 0.022), while no significant differences were found compared to ASL and the other diffusion models (all *p* > 0.05).

## Discussion

Accurately distinguishing between benign and malignant renal tumors and selecting the optimal treatment regimen is crucial to avoid unnecessary interventions and complications, as well as to reduce the risk of renal function impairment. Reliable noninvasive diagnostic methods are urgently needed. In our study, we first compared the diagnostic performance of six diffusion MRI models and assessed the additional value of combining ASL with diffusion models for differentiating benign and malignant renal tumors.

Our findings are as follows: (1) in differentiating ccRCC from non-ccRCC or benign renal tumors, ASL and all assessed diffusion models were effective with similar diagnostic performance, while combining ASL with diffusion models significantly improved the diagnostic performance; (2) in differentiating non-ccRCC from benign renal tumors, the IVIM model was more effective among ASL and the six diffusion models, whereas combining ASL with diffusion models had limited value in further improving diagnostic performance.

ccRCC is known for its high vascularity. Previous studies on ccRCC have demonstrated that tumor angiogenesis is associated with its prognosis, metastasis, and response to antiangiogenic therapy, highlighting the importance of evaluating renal tumor perfusion [26–30]. Our study firstly applied three-dimensional pseudocontinuous ASL to differentiate benign and malignant renal tumors, discovering that ccRCC showed the highest RBFmean and RBFpeak, followed by benign renal tumors, while non-ccRCC showed the lowest RBFmean and RBFpeak. This is not difficult to understand, since previous contrast-enhanced computed tomography or MRI studies have documented that ccRCC and oncocytoma are highly vascularized, papillary RCC is hypovascularized, and chromophobe RCC and fpAML’s vascularization falls between the two [31, 32]. Lanzman et al [19] found that oncocytoma showed the highest RBFmean and RBFpeak, which differed from our findings. This discrepancy may be because the benign renal tumors in our study included both oncocytoma (*n* = 8) and fpAML (*n* = 11). FpAML exhibits less enhancement than ccRCC, resulting in lower perfusion of benign renal tumors.

Diffusion MRI imaging is a powerful tool for exploring biological microstructural changes [33]. The *D* values (Mono_ADC, IVIM_D, DKI_MD, SEM_DDC, FROC_D, CTRW_D) are a core parameter in all diffusion models, which detect tissue water molecular diffusion characteristics under Gaussian or non-Gaussian diffusion behavior. Consistent with previous research, our study found that the *D* values of ccRCC were significantly higher than those of benign renal tumors and non-ccRCC [10, 12, 15]. This may be partly determined by their histopathological characteristics. ccRCC contained microcystic or macrocystic structures produced by dilated ternal acinar structures, as well as cystic and hemorrhagic areas [10], which allow for relatively less impeded water molecule diffusion. While fpAML was composed of different proportions of epithelial smooth muscle cells and tortuous thick-walled blood vessels [34], which restrict water diffusion. Conversely, Kilickesmez et al [35] found AML exhibited a higher ADC than ccRCC and noted a negative correlation between fat content and ADC values. This might be due to the different proportions of smooth muscle cells, tortuous blood vessels, and adipose tissue in AML. High-fat content tends to decrease ADC values, while high vascularity generates the opposite effect on ADC [15].

As advanced diffusion models, the CTRW, FROC, and SEM models can provide additional information about the water diffusion heterogeneity. The heterogeneity parameters (SEM_α, FROC_β, FROC_μ, CTRW_α, and CTRW_β) can directly reflect intravoxel diffusion heterogeneity in time and space, which is related to tissue complexity and microenvironment heterogeneity. To the best of our knowledge, there was a lack of study on the potential of the FROC and CTRW parameters in the discrimination of benign and malignant renal tumors. Our study found that ccRCC showed higher SEM_α, FROC_β, and CTRW_β compared to benign renal tumors, with no significant difference between ccRCC and non-ccRCC, consistent with Li et al [15]. One possible explanation is that fpAML may display more intravoxel heterogeneity than ccRCC, potentially linked to a higher degree of heterogeneous cells and tortuous vascular proliferation. DKI_MK represented the degree of water diffusion deviation from a Gaussian distribution and correlated positively with the nuclear-to-cytoplasm ratio [36]. Our study found that ccRCC showed lower DKI_MK compared to benign renal tumors and non-ccRCC, which was similar to Ding et al [10]. The increasing MK values likely are caused by greater viscosity and higher nuclear-to-cytoplasm ratio [36, 37].

Our study also found that the perfusion parameters D* and *f* are important in differentiating non-ccRCC from benign renal tumors. Compared with non-ccRCC, benign renal tumors showed a higher D* and *f*, aligned with Ding et al [38]. This can be explained by a previous study on computed tomography enhancement, which demonstrated that oncocytoma and fpAML are more enhanced than chromophobe RCC and papillary RCC [32]. Ding et al also found that ccRCC showed significantly higher D* and *f* compared with benign renal tumors, which conflicts with our results [10]. We found no difference in D* and *f* between ccRCC and benign renal tumors. In fact, the diagnostic value of D* and *f* is always controversial [39, 40]. The possible reasons are that (1) the selection and distribution of *b*-values are different, and (2) the sample composition is different. Therefore, further exploration is still needed.

Our study found that the six diffusion models showed similar diagnostic performance in differentiating ccRCC from non-ccRCC or benign renal tumors, while the IVIM model was more effective in differentiating non-ccRCC from benign renal tumors. Similar to our findings, Ding et al reported that the IVIM model showed better diagnostic performance than Mono_DWI in discriminating non-ccRCC from fpAML [38]. In contrast to our findings, Ding et al found that IVIM is the best in differentiating benign and malignant renal tumors [10]. This may be due to the difference in *b*-value distributions, scanners, field strengths, diffusion gradients, and so on, which will affect the reliability and reproducibility of outcomes [41]. A standardized DWI scanning protocol and *b*-value distribution will be crucial for validating these findings in the future, which will facilitate the clinical application of DWI models.

Receiver operating curve analysis supported the application of ASL in differentiating between benign and malignant renal tumors. More importantly, our study revealed that ASL achieved a higher AUC and lower AIC than diffusion models in distinguishing ccRCC from non-ccRCC, suggesting that ASL may provide more histopathological information about RCC subtypes. In our study, we also assessed the diagnostic performance of combining ASL with diffusion models for differentiating benign and malignant renal tumors. We found a significant improvement in the diagnostic accuracy and fitting performance compared to using ASL and diffusion models alone in differentiating ccRCC from non-ccRCC or benign renal tumors. This indicated that combining ASL with diffusion MRI models can provide additional value in the differential diagnosis of renal tumors. However, our observations did not show clear added value for combining ASL with diffusion MRI models in discrimination non-ccRCC from benign renal tumors. Therefore, large-scale prospective studies are needed to further validate our findings in the future.

Our study has several limitations. First, as a single-center, relatively small-sample, retrospective study, selection bias is unavoidable. Further multi-center studies with a large cohort are needed to validate the discriminative performance of these MRI-derived parameters. Second, in our study, renal tumors were roughly divided into three groups (ccRCC, non-ccRCC, and benign renal tumors), ignoring the potential differences in microstructural characteristics among different histological subtypes. As the sample size increases, we will refine the classification and further investigate the differences between different histopathological subtypes in the future. Third, we only assessed average metrics within the two-dimensional ROI, which may have limitations in capturing tumor internal heterogeneity. In the future, histogram analysis based on the whole tumor will be explored to validate these findings. Fourth, we acknowledge that having too many parameters in our study may increase the risk of model overfitting, potentially reducing the model’s generalizability and predictive accuracy. To minimize the risk, we used AIC to balance model complexity with predictive ability. Future research should validate the models’ generalizability by increasing sample sizes and conducting cross-validation. Lastly, our study did not exclude lesions of large size, which could affect the generalizability of the findings, particularly in cases with smaller or more challenging lesions. In the future, lesions of different sizes should be divided into multiple subgroups to assess the impact of lesion size on the findings.

In conclusion, our preliminary study demonstrated that ASL and all assessed diffusion MRI models can be feasible tools for differentiating ccRCC from non-ccRCC or benign renal tumors, with similar diagnostic performance. Combining ASL with diffusion models can offer more comprehensive information and significantly improve the diagnostic accuracy in the differentiation of ccRCC, which is valuable for selecting the optimal treatment strategy. Additionally, the IVIM model is more effective for differentiating non-ccRCC from benign renal tumors, whereas combining ASL with diffusion MRI models has limited additional value in further improving diagnostic performance.

## Supplementary information

**Additional file 1:** **Table S1.** Clinicopathological characteristics of patients with renal tumors. **Table S2.** The interobserver agreements between two radiologists of the ASL and diffusion parameters.

## Data Availability

The datasets used and/or analyzed during the current study are available from the corresponding author upon reasonable request.

## Abbreviations

ADC: Apparent diffusion coefficient
AIC: Akaike information criterion
ASL: Arterial spin labeling
AUC: Area under the receiver operating characteristic curve
CI: Confidence interval
CTRW: Continuous-time random walk
DKI: Diffusion kurtosis imaging
DWI: Diffusion-weighted imaging
fpAML: Fat-poor angiomyolipoma
FROC: Fractional-order calculus
IVIM: Intravoxel incoherent motion
MRI: Magnetic resonance imaging
RBF: Renal blood flow
ROI: Regions of interest
SEM: Stretched exponential model

## References

[1] Bukavina L, Bensalah K, Bray F et al (2022) Epidemiology of renal cell carcinoma: 2022 update. Eur Urol 82:529–542. 10.1016/j.eururo.2022.08.019 36100483 10.1016/j.eururo.2022.08.019

[2] Hollingsworth JM, Miller DC, Daignault S, Hollenbeck BK (2006) Rising incidence of small renal masses: a need to reassess treatment effect. J Natl Cancer Inst 98:1331–1334. 10.1093/jnci/djj362 16985252 10.1093/jnci/djj362

[3] Campbell SC, Novick AC, Belldegrun A et al (2009) Guideline for management of the clinical T1 renal mass. J Urol 182:1271–1279. 10.1016/j.juro.2009.07.004 19683266 10.1016/j.juro.2009.07.004

[4] Ljungberg B, Bensalah K, Canfield S et al (2015) EAU guidelines on renal cell carcinoma: 2014 update. Eur Urol 67:913–924. 10.1016/j.eururo.2015.01.005 25616710 10.1016/j.eururo.2015.01.005

[5] Schieda N, Davenport MS, Silverman SG et al (2023) Multicenter evaluation of multiparametric MRI clear cell likelihood scores in solid indeterminate small renal masses. Radiology 306:e239001. 10.1148/radiol.239001 36803006 10.1148/radiol.239001

[6] Jewett MA, Mattar K, Basiuk J et al (2011) Active surveillance of small renal masses: progression patterns of early stage kidney cancer. Eur Urol 60:39–44. 10.1016/j.eururo.2011.03.030 21477920 10.1016/j.eururo.2011.03.030

[7] Ball MW, Bezerra SM, Gorin MA et al (2015) Grade heterogeneity in small renal masses: potential implications for renal mass biopsy. J Urol 193:36–40. 10.1016/j.juro.2014.06.067 24960470 10.1016/j.juro.2014.06.067

[8] Marconi L, Dabestani S, Lam TB et al (2016) Systematic review and meta-analysis of diagnostic accuracy of percutaneous renal tumour biopsy. Eur Urol 69:660–673. 10.1016/j.eururo.2015.07.072 26323946 10.1016/j.eururo.2015.07.072

[9] Roussel E, Capitanio U, Kutikov A et al (2022) Novel imaging methods for renal mass characterization: a collaborative review. Eur Urol 81:476–488. 10.1016/j.eururo.2022.01.040 35216855 10.1016/j.eururo.2022.01.040 PMC9844544

[10] Ding Y, Tan Q, Mao W et al (2019) Differentiating between malignant and benign renal tumors: do IVIM and diffusion kurtosis imaging perform better than DWI? Eur Radiol 29:6930–6939. 10.1007/s00330-019-06240-6 31161315 10.1007/s00330-019-06240-6

[11] Li S, He K, Yuan G et al (2023) WHO/ISUP grade and pathological T stage of clear cell renal cell carcinoma: value of ZOOMit diffusion kurtosis imaging and chemical exchange saturation transfer imaging. Eur Radiol 33:4429–4439. 10.1007/s00330-022-09312-2 36472697 10.1007/s00330-022-09312-2

[12] Zhang J, Suo S, Liu G et al (2019) Comparison of monoexponential, biexponential, stretched-exponential, and kurtosis models of diffusion-weighted imaging in differentiation of renal solid masses. Korean J Radiol 20:791–800. 10.3348/kjr.2018.0474 30993930 10.3348/kjr.2018.0474 PMC6470087

[13] Tang L, Zhou XJ (2019) Diffusion MRI of cancer: from low to high *b*-values. J Magn Reson Imaging 49:23–40. 10.1002/jmri.26293 30311988 10.1002/jmri.26293 PMC6298843

[14] Le Bihan D, Breton E, Lallemand D, Aubin ML, Vignaud J, Laval-Jeantet M (1988) Separation of diffusion and perfusion in intravoxel incoherent motion MR imaging. Radiology 168:497–505. 10.1148/radiology.168.2.3393671 3393671 10.1148/radiology.168.2.3393671

[15] Li H, Liang L, Li A et al (2017) Monoexponential, biexponential, and stretched exponential diffusion-weighted imaging models: quantitative biomarkers for differentiating renal clear cell carcinoma and minimal fat angiomyolipoma. J Magn Reson Imaging 46:240–247. 10.1002/jmri.25524 27859853 10.1002/jmri.25524

[16] Alsop DC, Detre JA (1998) Multisection cerebral blood flow MR imaging with continuous arterial spin labeling. Radiology 208:410–416. 10.1148/radiology.208.2.9680569 9680569 10.1148/radiology.208.2.9680569

[17] Buchanan CE, Cox EF, Francis ST (2018) Evaluation of 2D imaging schemes for pulsed arterial spin labeling of the human kidney cortex. Diagnostics. 10.3390/diagnostics8030043 10.3390/diagnostics8030043 PMC6165477 29958409

[18] Pedrosa I, Rafatzand K, Robson P et al (2012) Arterial spin labeling MR imaging for characterisation of renal masses in patients with impaired renal function: initial experience. Eur Radiol 22:484–492. 10.1007/s00330-011-2250-z 21877173 10.1007/s00330-011-2250-z

[19] Lanzman RS, Robson PM, Sun MR et al (2012) Arterial spin-labeling MR imaging of renal masses: correlation with histopathologic findings. Radiology 265:799–808. 10.1148/radiol.12112260 23047841 10.1148/radiol.12112260 PMC3504320

[20] Tanaka F, Umino M, Maeda M et al (2022) Pseudocontinuous arterial spin labeling: clinical applications and ysefulness in head and neck entities. Cancers (Basel). 10.3390/cancers14163872 10.3390/cancers14163872 PMC9405982 36010866

[21] Kordbacheh H, Seethamraju RT, Weiland E et al (2019) Image quality and diagnostic accuracy of complex-averaged high *b* value images in diffusion-weighted MRI of prostate cancer. Abdom Radiol (NY) 44:2244–2253. 10.1007/s00261-019-01961-0 30838425 10.1007/s00261-019-01961-0

[22] Hu L, Zhou DW, Fu CX et al (2021) Advanced zoomed diffusion-weighted imaging vs. full-field-of-view diffusion-weighted imaging in prostate cancer detection: a radiomic features study. Eur Radiol 31:1760–1769. 10.1007/s00330-020-07227-4 32935192 10.1007/s00330-020-07227-4

[23] Lu F, Yang J, Yang S et al (2021) Use of three-dimensional arterial spin labeling to evaluate renal perfusion in patients with chronic kidney disease. J Magn Reson Imaging 54:1152–1163. 10.1002/jmri.27609 33769645 10.1002/jmri.27609

[24] Shirvani S, Tokarczuk P, Statton B et al (2019) Motion-corrected multiparametric renal arterial spin labelling at 3 T: reproducibility and effect of vasodilator challenge. Eur Radiol 29:232–240. 10.1007/s00330-018-5628-3 29992384 10.1007/s00330-018-5628-3 PMC6291439

[25] Pan W (2001) Akaike’s information criterion in generalized estimating equations. Biometrics 57:120–125. 10.1111/j.0006-341x.2001.00120.x 11252586 10.1111/j.0006-341x.2001.00120.x

[26] Mertz KD, Demichelis F, Kim R et al (2007) Automated immunofluorescence analysis defines microvessel area as a prognostic parameter in clear cell renal cell cancer. Hum Pathol 38:1454–1462. 10.1016/j.humpath.2007.05.017 17889675 10.1016/j.humpath.2007.05.017

[27] Panebianco V, Iacovelli R, Barchetti F et al (2013) Dynamic contrast-enhanced magnetic resonance imaging in the early evaluation of anti-angiogenic therapy in metastatic renal cell carcinoma. Anticancer Res 33:5663–5666 24324114

[28] Minardi D, Lucarini G, Filosa A et al (2008) Prognostic role of tumor necrosis, microvessel density, vascular endothelial growth factor and hypoxia inducible factor-1alpha in patients with clear cell renal carcinoma after radical nephrectomy in a long term follow-up. Int J Immunopathol Pharmacol 21:447–455. 10.1177/039463200802100225 18547492 10.1177/039463200802100225

[29] de Bazelaire C, Alsop DC, George D et al (2008) Magnetic resonance imaging-measured blood flow change after antiangiogenic therapy with PTK787/ZK 222584 correlates with clinical outcome in metastatic renal cell carcinoma. Clin Cancer Res 14:5548–5554. 10.1158/1078-0432.Ccr-08-0417 18765547 10.1158/1078-0432.CCR-08-0417

[30] Schor-Bardach R, Alsop DC, Pedrosa I et al (2009) Does arterial spin-labeling MR imaging-measured tumor perfusion correlate with renal cell cancer response to antiangiogenic therapy in a mouse model? Radiology 251:731–742. 10.1148/radiol.2521081059 19474376 10.1148/radiol.2521081059 PMC2687534

[31] Sun MR, Ngo L, Genega EM et al (2009) Renal cell carcinoma: dynamic contrast-enhanced MR imaging for differentiation of tumor subtypes-correlation with pathologic findings. Radiology 250:793–802. 10.1148/radiol.2503080995 19244046 10.1148/radiol.2503080995

[32] Zhang J, Lefkowitz RA, Ishill NM et al (2007) Solid renal cortical tumors: differentiation with CT. Radiology 244:494–504. 10.1148/radiol.2442060927 17641370 10.1148/radiol.2442060927

[33] Mao C, Hu L, Jiang W et al (2024) Discrimination between human epidermal growth factor receptor 2 (HER2)-low-expressing and HER2-overexpressing breast cancers: a comparative study of four MRI diffusion models. Eur Radiol 34:2546–2559. 10.1007/s00330-023-10198-x 37672055 10.1007/s00330-023-10198-x

[34] Humphrey PA, Moch H, Cubilla AL, Ulbright TM, Reuter VE (2016) The 2016 WHO classification of tumours of the urinary system and male genital organs-part B: prostate and bladder tumours. Eur Urol 70:106–119. 10.1016/j.eururo.2016.02.028 26996659 10.1016/j.eururo.2016.02.028

[35] Kilickesmez O, Inci E, Atilla S et al (2009) Diffusion-weighted imaging of the renal and adrenal lesions. J Comput Assist Tomogr 33:828–833. 10.1097/RCT.0b013e31819f1b83 19940645 10.1097/RCT.0b013e31819f1b83

[36] Dai Y, Yao Q, Wu G et al (2016) Characterization of clear cell renal cell carcinoma with diffusion kurtosis imaging: correlation between diffusion kurtosis parameters and tumor cellularity. NMR Biomed 29:873–881. 10.1002/nbm.3535 27119793 10.1002/nbm.3535

[37] Fu J, Ye J, Zhu W, Wu J, Chen W, Zhu Q (2021) Magnetic resonance diffusion kurtosis imaging in differential diagnosis of benign and malignant renal tumors. Cancer Imaging 21:6. 10.1186/s40644-020-00369-0 33413681 10.1186/s40644-020-00369-0 PMC7791668

[38] Ding Y, Zeng M, Rao S, Chen C, Fu C, Zhou J (2016) Comparison of biexponential and monoexponential model of diffusion-weighted imaging for distinguishing between common renal cell carcinoma and fat poor angiomyolipoma. Korean J Radiol 17:853–863. 10.3348/kjr.2016.17.6.853 27833401 10.3348/kjr.2016.17.6.853 PMC5102913

[39] Chandarana H, Lee VS, Hecht E, Taouli B, Sigmund EE (2011) Comparison of biexponential and monoexponential model of diffusion weighted imaging in evaluation of renal lesions: preliminary experience. Invest Radiol 46:285–291. 10.1097/RLI.0b013e3181ffc485 21102345 10.1097/RLI.0b013e3181ffc485

[40] Rheinheimer S, Stieltjes B, Schneider F et al (2012) Investigation of renal lesions by diffusion-weighted magnetic resonance imaging applying intravoxel incoherent motion-derived parameters-initial experience. Eur J Radiol 81:e310–e316. 10.1016/j.ejrad.2011.10.016 22104090 10.1016/j.ejrad.2011.10.016

[41] Jiang YL, Li J, Zhang PF et al (2024) Staging liver fibrosis with various diffusion-weighted magnetic resonance imaging models. World J Gastroenterol 30:1164–1176. 10.3748/wjg.v30.i9.1164 38577177 10.3748/wjg.v30.i9.1164 PMC10989501

