# Supplementary material for: Exploring the value of arterial spin labeling and six diffusion MRI models in differentiating solid benign and malignant renal tumors
Source: Eur Radiol Exp. 2024 Dec 5;8:135. doi: 10.1186/s41747-024-00537-y (PMC11621297; doi:10.1186/s41747-024-00537-y)
Supplement: Supplementary file 1 — Additional file 1: Table S1. Clinicopathological characteristics of patients with renal tumors. Table S2. The interobserver agreements between two radiologists of the ASL and diffusion parameters. [file 41747_2024_537_MOESM1_ESM.docx]

**Exploring the value of arterial spin labeling and six diffusion MRI**

**models in differentiating solid benign and malignant renal tumors**

**ELECTRONIC SUPPLEMENTARY MATERIAL**

**Appendix S1**

The post-progression software BoDiLab developed by ZhongYing was based on Python 3.8, which has been applied in previous studies [1-4]. A Gaussian filter was first applied to all ZOOMit DWI images with a sigma of 0.5 to increase the signal-to-noise ratio. Then, the parameters maps for the six models were calculated using the corresponding formulas.

For the **Monoexponential** model, a two-variable linear least-squares method was used here to fit the diffusion images voxel-by-voxel with b-values of 0 and 800 s/mm², and to calculate pixel-wise ADC (Mono_ADC) maps. The formula was as follows [5]:

Sb = S0 exp(−b ∙ ADC)

where S0 is the signal intensity (SI) when b=0 s/mm2, Sb is the SI at a given b value, ADC is the apparent diffusion coefficient.

For the **biexponential** model [5] (b=0, 20, 50, 80, 100, 200, 500, and 800s/mm2) Sb/S0 = (1 − f) exp(−b ∙ D) + f exp[−b(D∗ + D)]

where D is the true diffusion coefficient representing pure molecular diffusion, D* is the pseudo- diffusion coefficient related to water movement in the microcirculation, f is the perfusion fraction. A segmented fitting algorithm was used for the IVIM model, which has been applied in previous studies
[6-8]. Specific steps include (1) IVIM_D was firstly estimated using b-value above a threshold based on a linear fitting and ignoring perfusion compartment; (2) IVIM_f was calculated by comparing measured b = 0 signal and extrapolated b = 0 signal, based on the acquired IVIM_D in first step and the conventional Mono-exponential model; (3) IVIM_D* could be fitted using a linear form by fixing the IVIM_D and IVIM_f in IVIM model. The b value threshold at 200 was used, which is similar in previous study and could remove most of the perfusion signal.

Eur Radiol Exp (2024) Gao M, Li S, Yuan G, et al.

For the **DKI** model, a three-variable linear least-square method was used here to fit the diffusion images voxel-by-voxel with b-values of 0, 200, 500, 1000, 1500, and 2000s/mm2, and to calculate

pixel-wise kurtosis (DKI_MK) and diffusivity (DKI_MD) maps. The formula was as follows [5]:Sb = S0 exp(−b ∙ MD + b2 ∙ MD2 ∙ MK/6)

where MD (mean diffusivity) represents the ADC after non-Gaussian deviation modification, MK
(mean kurtosis) represents the degree of deviation from Gaussian distribution.

The SEM, FROC, and CTRW model were calculated by nonlinear fitting methods based on Levenberg-Marquardt algorithm with b-values of 0, 200, 500, 1000, 1500, and 2000s/mm2 [3,9], the corresponding formulas were as follows:

For the **SEM** model [5]

Sb = S0 exp[(−b ∙ DDC)α]

where α is intravoxel heterogeneity index, DDC is the distributed diffusion coefficient.

For the **FROC** model [9]

Sb = S0 exp [−Dμ2(β−1)(γGdδ)2β(∆ − $\frac{\text{2β}\text{ }\text{-}\text{ }\text{1}}{\text{2β}\text{ }\text{+}\text{ }\text{1}}$ δ)]

where Gd and δ represent the amplitude and pulse width of the diffusion gradient, respectively. Δ represents the gradient lobe separation. In this model, the diffusion coefficient (D) and the fractional order derivative in space (β) represent the spatial diffusion heterogeneity of water molecule motion.
μ is a spatial diffusion constant.

For the **CTRW** model [10]

Sb = S0Eα[(−b ∙ D)β]

where Eα represents the order-α Mittag-Leffler function, α represents the temporal diffusion heterogeneity, and β represents the spatial diffusion heterogeneity, D is the anomalous diffusion coefficient.

Eur Radiol Exp (2024) Gao M, Li S, Yuan G, et al.

**Appendix S2**

Quantitative renal blood flow (**RBF**) maps were generated according to the following formula [11] :

M0 −∆t −(t − τ − ∆t) −τ

∆M(t) = 2 f T
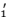
α exp ( ) ∙ exp ( ) ∙ (1 − exp ( ))

T
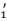


λ

T1,blood

T
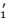


where M0 represents the equilibrium magnetization intensity of the tissue, λ represents the blood- tissue water partition coefficient, which was given as 0.9 mL/100 g, f represents the perfusion rate, with the unit of mL/100g/min. α represents the inversion efficiency, which was 0.98. ∆t

time of labeled blood, which was assumed to be as 750 msec,

the blood at 3.0 Tesla assumed to be as 1.2 sec. τ represents labeling time, both post- labeling delay and the labeling duration were 1500 msec.

T1,blood represents the T1 value of

with = +

1 1 f

is the arrival

15: 1387021.

100−107.

T
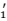
 T1 λ

105:

**Reference**

1. Han L, Yang J, Yuan C et al (2024) Assessing brain microstructural changes in chronic kidney disease: a diffusion imaging study using multiple models. Front Neurol

<https://doi.org/10.3389/fneur.2024.1387021>

1. Jiang Y, Fan F, Zhang P et al (2024) Staging liver fibrosis by a continuous-time random-walk

diffusion model. Magn

Reson

Imaging

<https://doi.org/10.1016/j.mri.2023.11.009>

1. Xie J, Li C, Chen Y et al (2023) Potential value of the stretched exponential and fractional order calculus model in discriminating between hepatocellular carcinoma and intrahepatic cholangiocarcinoma: an animal experiment of orthotopic xenograft nude mice. Curr Med Imaging. <https://doi.org/10.2174/1573405619666230322123117>
2. Jiang YL, Li J, Zhang PF et al (2024) Staging liver fibrosis with various diffusion-weighted magnetic resonance imaging models. World J Gastroenterol 30: 1164−1176.

<https://doi.org/10.3748/wjg.v30.i9.1164>

1. Bai Y, Lin Y, Tian J et al (2016) Grading of gliomas by using monoexponential, biexponential, and stretched exponential diffusion-weighted MR imaging and diffusion kurtosis MR imaging. Radiology 278: 496−504. <https://doi.org/10.1148/radiol.2015142173>
2. Zhang MC, Li XH, Huang SY et al (2019) IVIM with fractional perfusion as a novel biomarker for detecting and grading intestinal fibrosis in Crohn's disease. Eur Radiol 29: 3069−3078.

<https://doi.org/10.1007/s00330-018-5848-6>

1. Xiang Z, Ai Z, Liang J, Li G, Zhu X,Yan X (2018) Evaluation of regional variability and measurement reproducibility of intravoxel incoherent motion diffusion weighted imaging using a cardiac stationary phase based ECG trigger method. Biomed Res Int 2018: 4604218.

<https://doi.org/10.1155/2018/4604218>

1. Lu B, Yang X, Xiao X, Chen Y, Yan X,Yu S (2018) Intravoxel incoherent motion diffusion-

Eur Radiol Exp (2024) Gao M, Li S, Yuan G, et al.

weighted imaging of primary rectal carcinoma: correlation with histopathology. Med Sci Monit 24: 2429−2436. <https://doi.org/10.12659/msm.908574>

1. Sui Y, Wang H, Liu G et al (2015) Differentiation of low- and high-grade pediatric brain tumors with high b-value diffusion-weighted MR imaging and a fractional order calculus model. Radiology 277: 489−496. <https://doi.org/10.1148/radiol.2015142156>
2. Zhong Z, Merkitch D, Karaman MM et al (2019) High-spatial-resolution diffusion MRI in parkinson disease: lateral asymmetry of the substantia nigra. Radiology 291: 149−157.

<https://doi.org/10.1148/radiol.2019181042>

1. Robson PM, Madhuranthakam AJ, Dai W, Pedrosa I, Rofsky NM,Alsop DC (2009) Strategies for reducing respiratory motion artifacts in renal perfusion imaging with arterial spin labeling. Magn Reson Med 61: 1374−1387. <https://doi.org/10.1002/mrm.21960>

Table S1 Clinicopathological characteristics of patients with renal tumors.

| Parameter | ccRCC | Non-ccRCC | Benign | *p* value |
| --- | --- | --- | --- | --- |
| No. of patients Age (years)a | 48  55.92±11.62 | 22  52.36±14.72 | 19  47.63±14.94 | 0.069 |
| Gender |  |  |  | *0.011* |
| Male | 27(56.25%) | 11(50.00%) | 3(15.79%) |  |
| Female BMI (kg/m²) a | 21(43.75%) 24.08±3.09 | 11(50.00%) 23.55±2.12 | 16(84.21%) 24.14±3.32 | 0.750 |
| Maximum tumor diameter(cm)a | 5.60±2.71 | 5.71±3.15 | 4.03±2.90 | *0.021** |
| Location |  |  |  | 0.727 |
| Left | 24(50.00%) | 12(54.55%) | 8(42.11%) |  |
| Right | 24(50.00%) | 10(45.45%) | 11(57.89%) |  |

WHO/ISUP grade

| I | 10(20.83%) |
| --- | --- |
| II | 24(50%) |
| III | 10(20.83%) |
| IV | 4(8.33%) |

a Data are expressed as mean ± standard deviation (SD), others are expressed as n (%).

*Kruskal-Wallis H test with Bonferroni correction, others are one-way ANOVA or Chi-square test. The *p* values in italics are of statistical significance.

Eur Radiol Exp (2024) Gao M, Li S, Yuan G, et al.

Table S2 The interobserver agreements between two radiologists of the ASL and diffusion parameters.

| Parameters | ICC (95% CI) | *p* |
| --- | --- | --- |
| RBFmean | 0.983(0.974-0.989) | <0.001 |
| RBFpeak | 0.993(0.989-0.995) | <0.001 |
| Mono ADC | 0.991(0.986-0.994) | <0.001 |
| IVIM D | 0.991(0.987-0.994) | <0.001 |
| IVIM D* | 0.935(0.902-0.957) | <0.001 |
| IVIM f | 0.950(0.925-0.967) | <0.001 |
| DKI MD | 0.988(0.981-0.992) | <0.001 |
| DKI MK | 0.980(0.970-0.987) | <0.001 |
| SEM α | 0.948(0.922-0.966) | <0.001 |
| SEM DDC | 0.981(0.972-0.988) | <0.001 |
| FROC β | 0.920(0.880-0.946) | <0.001 |
| FROC D | 0.991(0.986-0.994) | <0.001 |
| FROC μ | 0.991(0.987-0.994) | <0.001 |
| CTRW α | 0.937(0.906-0.958) | <0.001 |
| CTRW β | 0.926(0.889-0.951) | <0.001 |
| CTRW D | 0.989(0.983-0.993) | <0.001 |

*ADC* apparent diffusion coefficient, *CI* confdence intervals, *CTRW*  *α* temporal diffusion heterogeneity index, *CTRW*  *β* spatial diffusion heterogeneity index, *DKI_MD* mean diffusivity, *DKI_MK* mean kurtosis, *FROC_β* spatial diffusion heterogeneity index, *FROC_μ* spatial diffusion constant, *ICC* intraclass correlation coefcient, *IVIM_D* true diffusivity, *IVIM_D** pseudo-diffusion coefficient, *IVIM_f* perfusion fraction, *RBF* renal blood flow, *SEM_DDC· FROC_D· CTRW_D* diffusion coefficient, *SEM_α* intravoxel heterogeneity index,

The unit for “RBF” is ml/100g/min. DKI MK, SEM α, FROC β, FROC μ, CTRW α, CTRW_β have no unit. Mono_ADC, IVIM_D, IVIM_D*, DKI_MD, SEM_DDC, FROC_D, CTRW_D values are presented in × 10−3 mm2/s, IVIM_f is presented in percentage.

Eur Radiol Exp (2024) Gao M, Li S, Yuan G, et al.
